# An In Situ Sustained-Release Chitosan Hydrogel to Attenuate Renal Fibrosis by Retaining Klotho Expression

**DOI:** 10.34133/bmr.0099

**Published:** 2024-10-24

**Authors:** Chenyang Li, Shuai Wang, Chenghui Liao, Ying Li, Yunfeng Zhou, Haiqiang Wu, Wei Xiong

**Affiliations:** ^1^ School of Pharmacy, Shenzhen University Medical School, Shenzhen University, Shenzhen 518055, China.; ^2^ School of Basic Medical Sciences, Shenzhen University Medical School, Shenzhen University, Shenzhen 518055, China.

## Abstract

Klotho (KLO) is an anti-fibrotic protein expressed in the kidneys and has been decreasing in the development of renal fibrosis (RF). However, restoring the decline in KLO levels remains a great challenge during RF treatment. Herein, an injectable KLO-loaded chitosan (CS) hydrogel (KLO-Gel) is designed to achieve localized and prolonged release of KLO in the RF treatment. KLO-Gel was prepared by cross-linking CS with β-glycerophosphate (β-GP), followed by rapid (within 3 min) thermosensitive gelation at 37 °C. Furthermore, KLO-Gel exhibited a slow and sustained release (over 14 d) of KLO both in PBS and in the kidneys of mice with unilateral ureter obstruction (UUO). A single local injection of KLO-Gel into the renal capsule of UUO mice was more effective at reducing RF (i.e., maintaining renal function and tissue structure, alleviating extracellular matrix accumulation, and inhibiting the TGF-β1/Smad2/3 signaling pathway) over a 14-d period than daily intraperitoneal injections of free KLO or captopril. Crucially, CS was found to induce endogenous KLO secretion, highlighting the added value of using CS in RF treatment. Overall, this study demonstrated that KLO-Gel enhanced the anti-fibrotic efficacy of KLO while minimizing its off-target toxicity, and its clinical potential awaits further validation.

## Introduction

Renal fibrosis (RF) is a chronic and progressive process, which occurs during normal aging and in chronic kidney disease (CKD), leading to the deterioration and eventual loss of kidney function [1,2]. RF and CKD affect 50% of older adults (aged >70 years) and 10% of the global population [3]. Treatment strategies for preventing and reversing RF, such as angiotensin-converting enzyme inhibition [4], angiotensin receptor blockade [5], and optimal blood pressure control [6], are limited. Moreover, the outcomes of these therapies remain poor, resulting in RF progression to end-stage renal disease, which ultimately requires dialysis or kidney transplantation.

Klotho (KLO), a membrane-bound protein predominantly expressed in the kidneys, exerts anti-fibrotic effects in the renal and cardiac tissues [7]. When the kidney is damaged, membrane-bound KLO (mKLO) is cleaved from the surface of renal epithelial cells and is released as soluble KLO (sKLO; 130 kDa). A pioneering study reported that KLO deficiency accelerated the progression of aging-related diseases in mice [8]. Crucially, both mKLO overexpression and sKLO supplementation via injection prevented RF progression [9,10]. Moreover, sKLO has been demonstrated to act as an anti-fibrotic agent via its direct inhibition of RF-related pathways, including transforming growth factor-β (TGF-β) [11], Wnt [12,13], and fibroblast growth factor 2 (FGF2) [14] signaling.

KLO supplementation and overexpression are 2 main approaches that have been used to attenuate RF development. For instance, the daily injection of recombinant KLO significantly improved renal function and histological damage in mice with unilateral ureteral obstruction (UUO) and ischemia–reperfusion injury (IRI) [15]. The intravenous injection of recombinant KLO, however, elicits severe side effects and has low kidney selectivity, which greatly restricts its clinical application. To date, a number of studies have managed to increase endogenous KLO expression by targeting the epigenetic regulation of its gene expression, for instance, using Rhein to demethylate the KLO promoter and then decreased RF in both the UUO and adenine-induced renal failure models [16]. In terms of chronic diseases such as RF, systemic administration often faces premature drug release and nonrenal distribution, resulting in poor therapeutic efficacy and off-target toxicity.

To realize KLO-based treatment against RF, an in situ injectable hydrogel—simultaneously allows kidney localized KLO delivery and KLO overexpression—is highly attractive. Chitosan (CS) is a natural polysaccharide, which consists of β-1,4-linked d-glucosamine units and has favorable biocompatibility and biodegradability [17,18]. After crosslinking with β-GP, CS can achieve sol–gel transition at body temperature [19]. The mechanical properties of CS hydrogels can be tailored by varying the concentration of CS [20]. Also, CS hydrogels have been demonstrated to control and/or delay drug release within the hydrogel matrix [21]. Due to these advantages, CS has been widely explored for biomedical applications. Although CS has been demonstrated to act a promising kidney-targeting carrier by ourselves and others [22,23], the potential of CS to alleviate RF as well as the underlining mechanisms are still unknown.

**Fig. 1.**
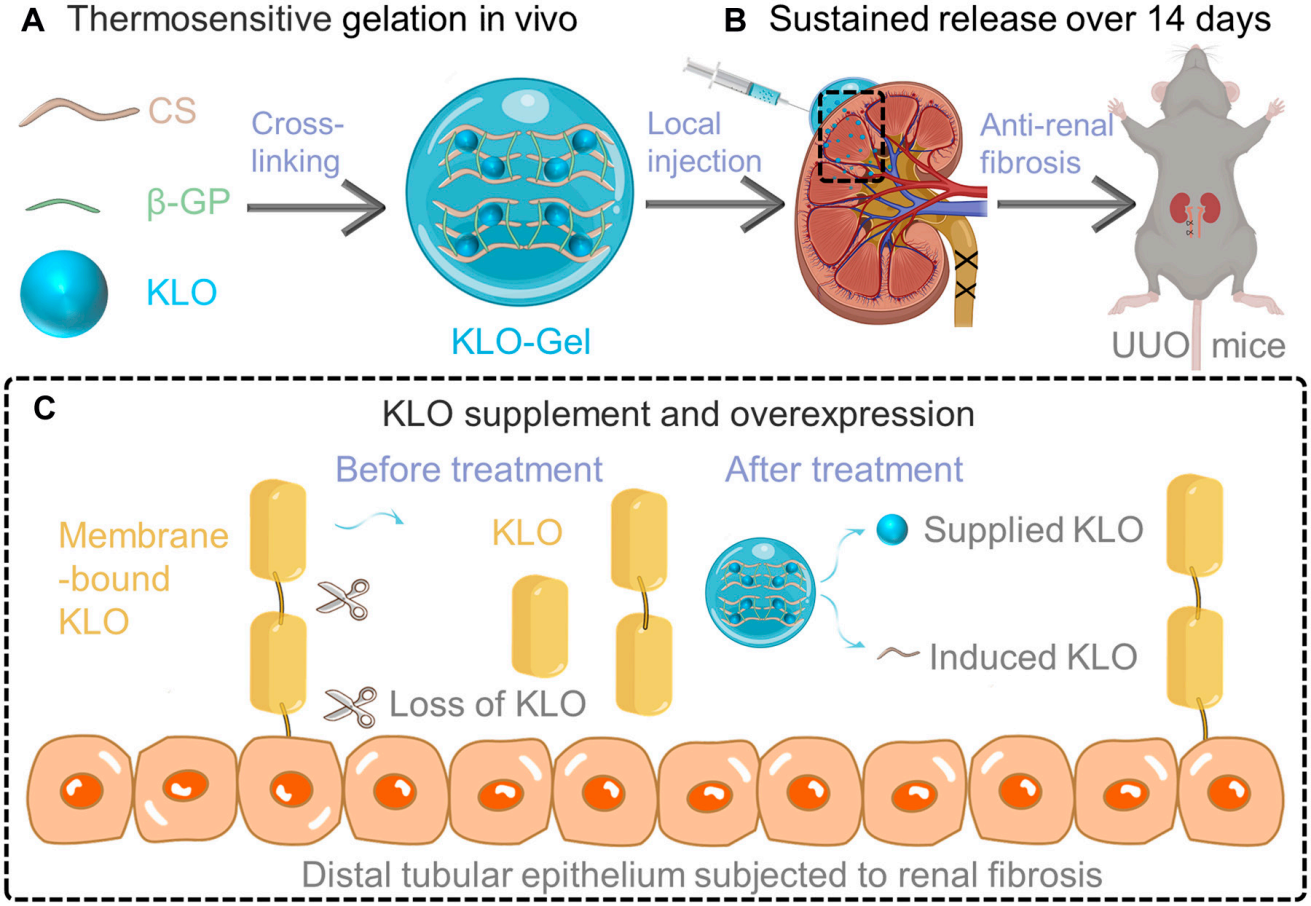
Schematic illustration of KLO-Gel for localized and extended release of KLO in the treatment of RF. (A) KLO-Gel is prepared by cross-linking CS and β-GP before performing thermosensitive gelation in vivo. (B) After delivery of KLO-Gel via local injection into the renal capsule of UUO mice, KLO release is sustained over 14 d. (C) The localized injection of KLO-Gel alleviates RF by increasing concentration of recombinant KLO in the kidney while also inducing endogenous KLO overexpression.

Overall, the aim of the present study was to design an injectable KLO-loaded CS hydrogel (KLO-Gel) to achieve the localized and prolonged release of KLO and demonstrate its feasibility as an RF treatment strategy (Fig. 1). After thermosensitive gelation and formulation optimization studies, the sustained-release manner of KLO-Gel was comprehensively investigated in vitro and in vivo. Furthermore, the therapeutic efficacy of KLO-Gel was evaluated in UUO mice and the effects of CS on inducing endogenous KLO were deeply investigated.

## Materials and Methods

### Chemicals, reagents, and animals

The following reagents and chemicals were used in this study: CS (molecular weight 20 kDa, deacetylation degree 85%) was bought from Haidebei Marine Bioengineering Co. Ltd. (Jinan, China); β-GP was sourced from Aladdin (Shanghai, China); and Cy5.5 and bovine serum albumin (BSA) were purchased from J&K Scientific Ltd. (Beijing, China). Recombinant mouse KLO (aa 35-982) was obtained from R&D Systems (Minneapolis, USA). All reagents and chemicals were of analytical grade.

The rat kidney cell line NRK-52E (passage numbers 5 to 10) was purchased from Zhong Qiao Xin Zhou Biotechnology Co. Ltd. (Shanghai, China) and cultured in Dulbecco’s modified Eagle’s medium (DMEM) (Gibco, Beijing, China) supplemented with 5% fetal bovine serum (FBS) (TransGen Biotech, China) and 1% penicillin/streptomycin (Gibco, USA) in an incubator (Thermo, USA) at 37 °C and 5% CO_2_. The cells were collected upon reaching 80% confluence, and the medium was changed every 48 h.

Male C57BL/6 (aged 8 weeks, 25 to 28 g) and BALB/c (aged 6 weeks, 22 to 25 g) mice were provided by Guangdong Medical Laboratory Animal Center (Foshan, China). Mice were maintained under standard conditions at 22 °C, humidity 60% ± 10%, and a 12-h light/dark cycle, with free access to fresh water and rodent chow. Mice were used in experiments after a 1-week adaptation to their new environment. All experimental protocols were performed in accordance with the National Research Council’s Guide for the Care and Use of Laboratory Animals and approved by the Animal Research Ethics Committee, Shenzhen University, Shenzhen, China.

### Preparation and characterization of KLO-Gel

#### Preparation of KLO-Gel

KLO-Gel was prepared using a previously published cross-linking method with minor modifications [19]. Briefly, the CS solution (2%, dissolved in 0.6% acetic acid, w/w) and β-GP solution [50%, dissolved in double-distilled water (ddH_2_O), w/w] were precooled on ice. Blank hydrogel (BLK-Gel; not loaded with recombinant KLO) was formed by mixing with CS and β-GP solutions at different volume ratios, including CS:β-GP = 2:1, 3:1, 4:1, and 5:1 (v/v), which were equal to the mass ratios of CS:β-GP = 1:12.5, 1:8.3, 1:6.25, and 1:5 (w/w), respectively. The thermosensitive gelation of BLK-Gel was performed at 37 °C for 30 min. The gelation status of BLK-Gel was observed after heating every 3 min for 30 min. KLO-Gel was prepared by adding the recombinant KLO (50 μg) before gelation. The pH of KLO-Gel was determined to be 7.0.

To better assess the in vitro and in vivo sustained release of KLO-Gel, fluorescent Cy5.5-BSA was applied and loaded into the hydrogel. The Cy5.5-BSA was synthesized according to a previously reported method [24] . The Cy5.5-BSA-Gel was prepared by the same protocol, except for the final Cy5.5-BSA concentration of 6.25 μg/ml. Similar to KLO-Gel, Cy5.5-BSA-Gel also performed rapid thermosensitive gelation at 37 °C (Fig. S1).

#### Characterization of KLO-Gel

The gelation kinetics of KLO-Gel were assessed using a time sweep model in an MCR92 advanced rotational rheometer (Anton Paar, Graz, Austria). The parallel plates were set at 1-Hz frequency, with 5% strain and a 50-min scanning time. The relationship between storage modulus (*G*′) and loss modulus (*G*″) with respect to time was obtained [25]. The morphology and structural characteristics of KLO-Gel were examined using an Apreo 2C scanning electron microscope (SEM) (Thermo Scientific, Waltham, MA, USA) as previously described [26]. CS powder and freeze-dried KLO-Gel were thinly sprinkled onto a metal stub and vacuum-coated with a thin layer of gold in an argon atmosphere, respectively. Then, these coated samples were examined at an acceleration voltage of 15 kV. The swelling ratio and degradation percentage of KLO-Gel were investigated as previously reported, with minor modifications [25].

### In vitro release assay

The in vitro release study was performed to determine the cumulative release of Cy5.5-BSA rather than that of KLO [27]. Cy5.5-BSA-Gel (1 ml, containing 6.25 μg of Cy5.5-BSA) was placed in a dialysis bag (with a molecular weight cutoff of 100 kDa). The dialysis bag was then placed in a dissolution cup containing 150 ml of phosphate-buffered saline (PBS) (pH 7.4) and incubated at 37 °C. At the indicated time points (0.25, 0.5, 1, 2, 4, 7, 11, and 14 d), 1 ml of the released sample was collected and replenished with an equal volume of fresh PBS. The cumulative release of Cy5.5-BSA was determined using a fluorescence spectrophotometer (Techcomp Ltd., Beijing, China) by the reported method as described previously [24].

### In vivo release assay

UUO surgery was performed in the male BALB/c mice via left ureter ligation. Then, a solution of Cy5.5-BSA-Gel (50 μl per mouse, containing 6.25 μg of Cy5.5-BSA) was injected into the renal capsule of the left kidney. The retention of Cy5.5-BSA-Gel in the left kidney was visualized using an In Vivo Imaging System (IVIS) spectrum imaging system (PerkinElmer, USA) [25]. All mice were sacrificed by CO_2_ inhalation on day 14, and their hearts, livers, spleens, lungs, and kidneys were collected and subjected to ex vivo fluorescence imaging.

### Anti-fibrotic effects in TGF-β1-treated NRK-52E cells

The cell model of TGF-β1-induced RF was established in NRK-52E cells as previously reported [28]. Briefly, NRK-52E cells were seeded at a density of 1 × 10^5^ cells/ml in 6-well plates and allowed to adhere overnight. The culture medium was changed to serum-free medium for 24 h, and then the cells were cotreated with TGF-β1 (10 ng/ml) (Sino Biological, Beijing, China) and free KLO (0.25 ng/ml), KLO-Gel (containing 0.25 ng/ml KLO), or BLK-Gel (150 μg/ml CS) for 48 h. The mRNA and protein expression levels of RF-related factors were detected by real-time polymerase chain reaction (PCR) and Western blotting, respectively, as previously reported [29].

### Assessing anti-fibrotic efficacy in UUO mice

#### UUO induction and treatment

The mouse model of UUO-induced RF was established in C57BL/6 mice as previously reported [23] on day 1. The UUO mice were then randomly divided into 5 groups (*n* = 6): (a) UUO (model group), (b) free KLO (10 μg/kg/day, intraperitoneally), (c) BLK-Gel (50 μl, local renal injection), (d) KLO-Gel (50 μl, containing 3.75 μg of KLO, local renal injection), and (e) captopril (10 mg/kg/day, intraperitoneally). BLK-Gel and KLO-Gel were injected into the renal capsule of the left kidney during UUO surgery (day 1). The mice in the free KLO and captopril groups were injected daily from day 1 until day 14.

Captopril was chosen as a positive drug in this study, and its dose was based on the previous studies and clinical use [30]. The mice in normal and model groups received an equal volume of PBS intraperitoneal injection daily. All mice were sacrificed by CO_2_ inhalation on day 15 after surgery; their blood and kidney samples were then collected. The left kidneys of mice were decapsulated, washed, and dissected; part of each kidney was fixed in 10% formalin, and the remaining part was stored at −80 °C to be used for Western blotting.

#### Kidney appearance evaluation

The kidneys harvested from all the mouse groups were compared and photographed to assess their appearance and general condition.

#### Analysis of renal function

Serum creatinine levels were determined using a commercial creatinine assay kit (Jiancheng, Nanjing, China). Serum blood urea nitrogen (BUN) levels were determined using a commercial urea nitrogen content assay kit (Solarbio, Beijing, China) as previously described [31].

#### Histopathology

The left kidneys of mice were excised, formalin fixed, and paraffin sectioned. After hematoxylin and eosin (H&E), Masson’s, and immunohistochemical staining as previously reported method [23], histological examination of the kidneys was assessed under a microscope (Olympus, Japan).

#### Western blotting

Kidney samples were prepared for Western blotting as described previously [23]. After isolating total protein from the kidney tissues, the samples were incubated with primary antibodies against fibronectin (1:1000, Proteintech, USA), E-cadherin (1:1000, Proteintech, USA), KLO (1:1000, Abcam, USA), Smad2/3 (1:1000, Cell Signaling Technology, USA), pSmad2/3 (1:1000, Cell Signaling Technology, USA), α-SMA (1:1000, Proteintech, USA), TGF-β1 (1:1000, Cell Signaling Technology, USA), and glyceraldehyde 3-phosphate dehydrogenase (GAPDH; 1:1000, Abbkine, USA). After incubation with a secondary antibody, the protein bands were imaged and quantified (Bio-Rad, USA).

### Assessing the effect of CS on KLO expression

The effect of CS on KLO expression was investigated in vitro and in vivo. First, the effect of CS on KLO expression was studied in TGF-β1-treated NRK-52E cells. As mentioned in the “Anti-fibrotic effects in TGF-β1-treated NRK-52E cells” section, after culture in serum-free medium for 24 h, the cells were cotreated with TGF-β1 and CS (75, 150, and 300 μg/ml) for another 48 h. The concentration of KLO secreted into the culture medium was determined using a KLO enzyme-linked immunosorbent assay (ELISA) assay kit (Tongwei Industry Co. Ltd., Shanghai, China). The serum and renal KLO levels of UUO mice exposed to various treatment conditions were measured using the same KLO ELISA assay kit.

### Safety and biocompatibility evaluation

The male BALB/c were anesthetized with 10% chloral hydrate (400 mg/kg, intraperitoneally), and their left kidneys were exposed through an incision to the lateral dorsal surface. After injecting KLO-Gel (50 μl, containing 3.75 μg of KLO) into the renal capsule of each left kidney, the incised dorsal surface was sutured. The normal mice in the control group received an equal volume of PBS intraperitoneal injection daily. All mice were sacrificed by CO_2_ inhalation on day 15 after surgery; their serum and organ samples were collected and stored at −80 °C. Various biochemical indicators such as liver enzyme activity and creatine kinase levels were determined in the sera of mice using the corresponding assay kits (Jiancheng Biotech. Co. Ltd., China). The mouse liver and kidney tissues were fixed in formalin and stained with H&E for histopathological observation.

### Statistical analysis

Data analysis was conducted using GraphPad Prism and ImageJ. The one-way analysis of variance (ANOVA) was used to determine the statistical significance of differences among the various treatment groups. The following *P* values were used as statistical significance thresholds: *P < 0.05, **P < 0.01, ***P < 0.001, ****P < 0.0001.

## Results

### Thermosensitive gelation

**Fig. 2.**
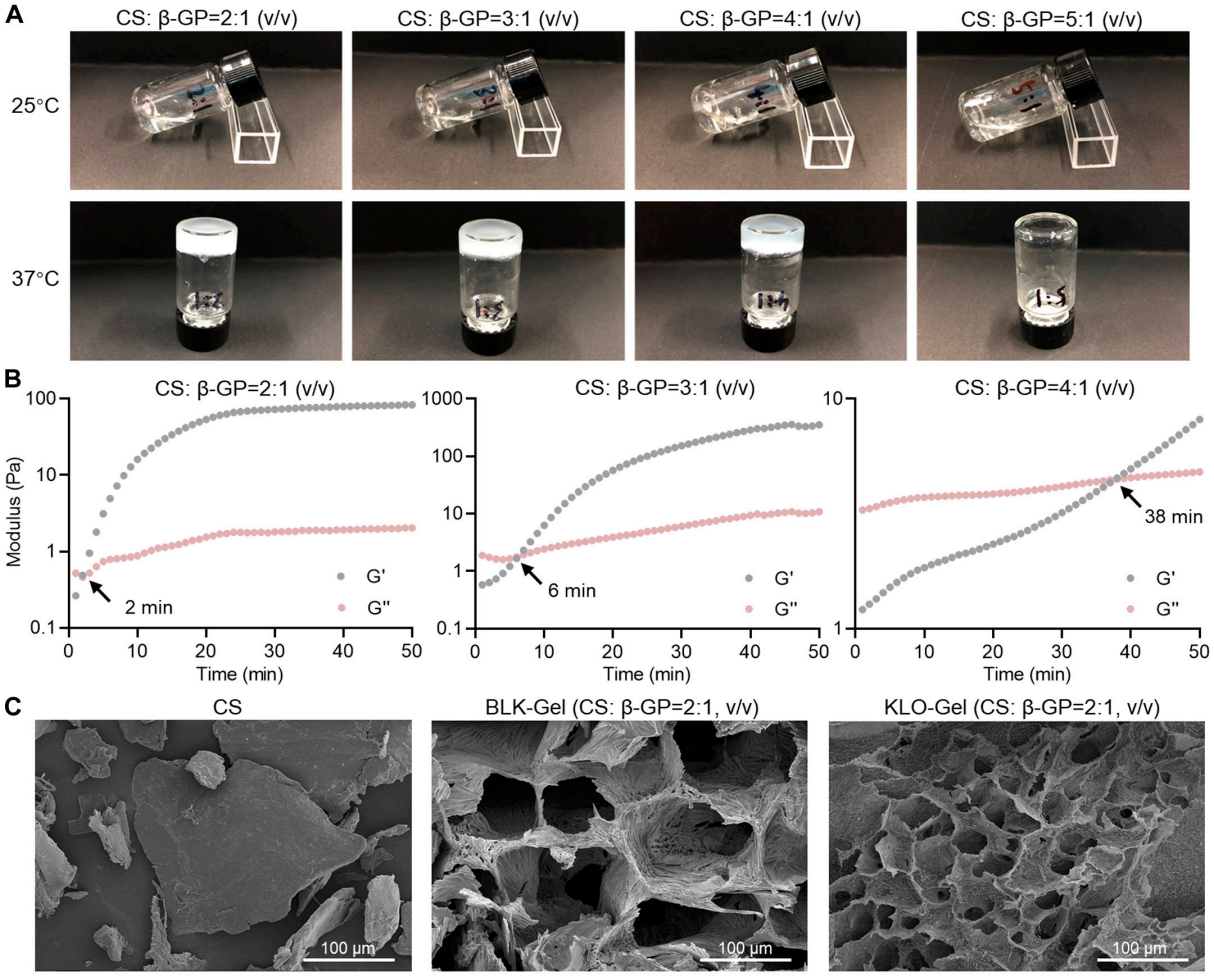
Thermosensitive gelation and characterization of KLO-Gel. (A) KLO-Gel was prepared using different CS to β-GP ratios at 25 °C before undergoing thermosensitive gelation at 37 °C. (B) Rheological characterization of KLO-Gel using dynamic time sweep tests performed at 37 °C. (C) Representative SEM images of lyophilized KLO-Gel; CS alone was used as a negative control.

To perform the thermosensitive gelation of KLO-Gel, CS (2%, dissolved in 0.6% acetic acid, w/w) was cross-linked with β-GP (50%, dissolved in ddH_2_O, w/w) at different ratios at 25 °C. Thermosensitive gelation was observed at all CS:β-GP ratios except for the formulation prepared with CS:β-GP = 5:1 (v/v) after incubating at 37 °C for 30 min (Fig. 2A). The gelation time increased with increasing CS:β-GP ratio; thus, the gelation times for the CS:β-GP = 2:1, 3:1, and 4:1 (v/v) formulations were 3, 5, and 30 min, respectively. In accordance, the sol–gel transition times for the 3 formulations in the strain oscillatory shear experiments were 2, 6, and 38 min, respectively, at 37 °C (Fig. 2B). To further confirm the presence of a fully cured hydrogel network, the microstructure of KLO-Gel was observed under a SEM after lyophilization. Unlike the CS alone, both KLO-Gel (CS:β-GP = 2:1, v/v) and BLK-Gel had a typical porous network structure (Fig. 2C). These results demonstrated that KLO-Gel was successfully prepared and had thermosensitive gelation properties.

### In vitro sustained release

**Fig. 3.**
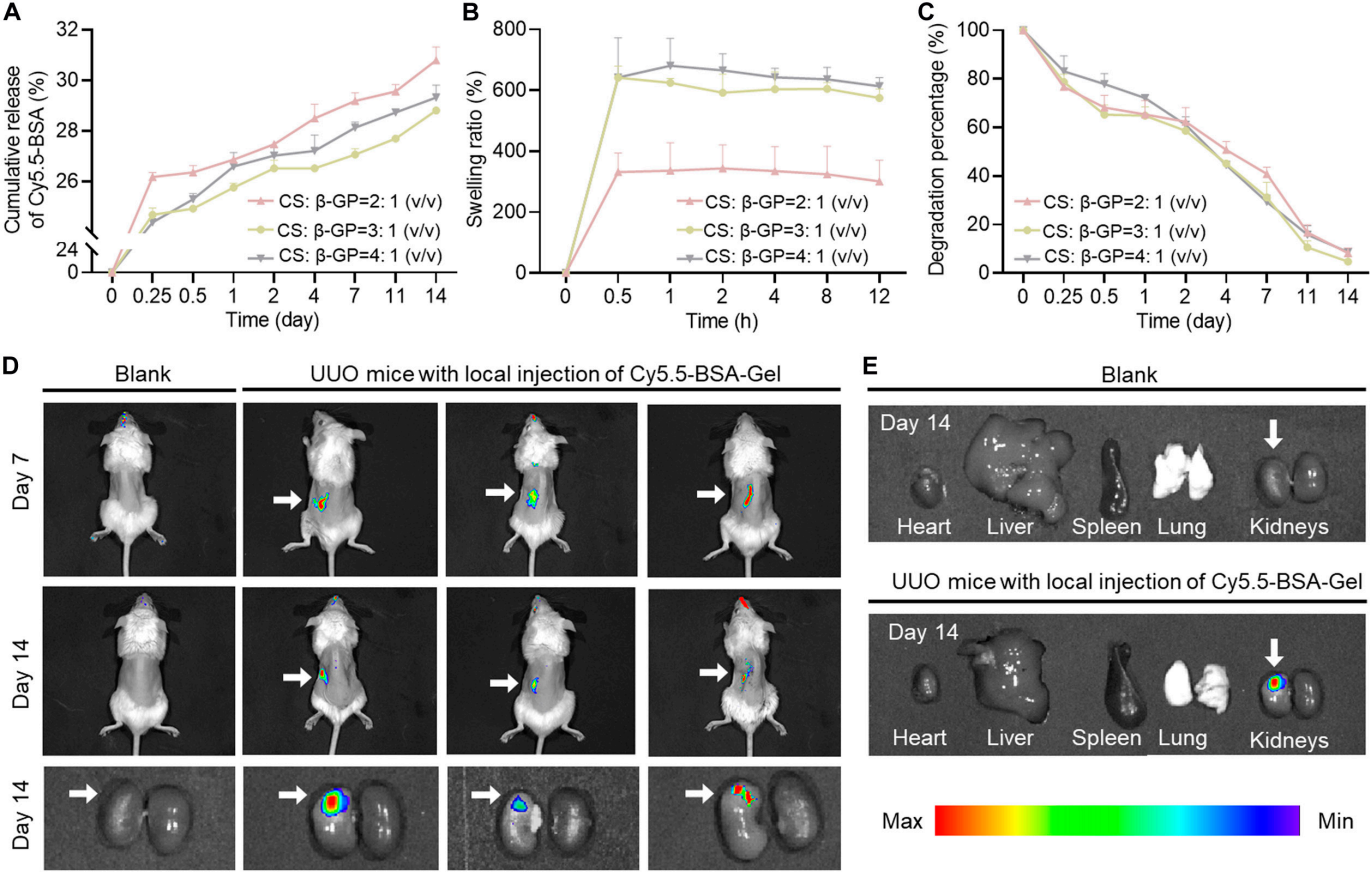
Monitoring the sustained-release properties of Cy5.5-BSA-Gel over 14 d. (A) In vitro release profiles of the 3 Cy5.5-BSA-Gel formulations in PBS. (B) The degree of swelling of the Cy5.5-BSA-Gel formulations was monitored in PBS over 12 h. (C) The degree of Cy5.5-BSA-Gel degradation was assessed in PBS over 14 d. (D) Representative in vivo fluorescent images taken after the local injection of Cy5.5-BSA-Gel into the renal capsule of the left kidney of each UUO mouse. (E) Representative images showing the biodistribution of Cy5.5-BSA in the various organs of UUO mice on day 14.

To visualize the ability of KLO-Gel to release KLO in vitro for extended periods of time, KLO was replaced by the fluorescent reagent Cy5.5-BSA in the KLO-Gel preparation to form Cy5.5-BSA-Gel. Cy5.5-BSA-Gel was subsequently used in the in vitro release experiments performed in PBS (pH 7.4). As expected, all the Cy5.5-BSA-Gel formulations (i.e., CS:β-GP = 2:1, 3:1, and 4:1, v/v) achieved a slow and sustained release of Cy5.5-BSA for 14 d; the cumulative release of Cy5.5-BSA was 31.2%, 27.9%, and 29.3% for the 3 formulations, respectively (Fig. 3A). Furthermore, the swelling and degradation properties of Cy5.5-BSA-Gel were investigated. The swelling ratio of Cy5.5-BSA-Gel prepared with CS:β-GP = 2:1, 3:1, and 4:1 (v/v) was determined to be 276.2%, 642.0%, and 681.2% in PBS, respectively (Fig. 3B). Meanwhile, all Cy5.5-BSA-Gel formulations presented a slow and steady degradation in PBS for 14 d (Fig. 3C). By day 14, the Cy5.5-BSA-Gel formulations prepared with CS:β-GP = 2:1, 3:1, and 4:1 (v/v) lost 91.7%, 95.2%, and 91.3% of their mass. These results indicate that the release of Cy5.5-BSA was mainly driven by diffusion and polymer erosion. Based on the thermosensitive gelation time (Table S1) and sustained-release capacity (Fig. 3A), the CS:β-GP = 2:1 (v/v) formulation was selected for use in the subsequent experiments.

### In vivo sustained release

To confirm whether Cy5.5-BSA-Gel also had favorable sustained-release characteristics in vivo, Cy5.5-BSA-Gel was injected into the renal capsule of the left kidney of each UUO mouse and visualized Cy5.5-BSA-derived fluorescence using the IVIS imaging system. As shown in Fig. 3D, obvious fluorescent signals were observed over the 14-d period, with a gradual decay in fluorescence intensity over time. Of note, the Cy5.5-BSA signals were localized to the left kidney, without spreading to other organs (Fig. 3E). These results demonstrate that Cy5.5-BSA-Gel achieved a localized, slow, and sustained release of its cargo in vivo.

### In vitro anti-fibrotic efficacy

**Fig. 4.**
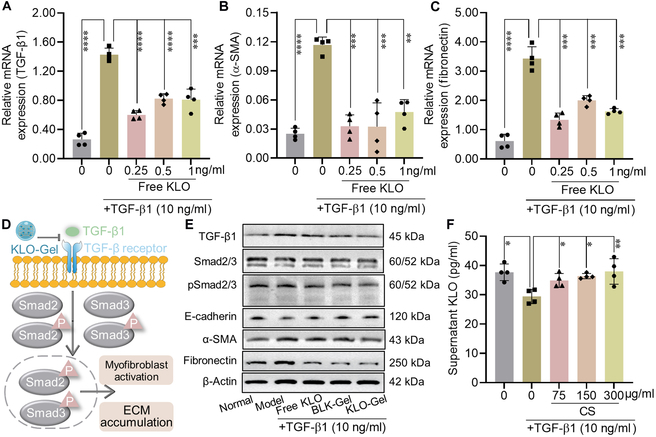
KLO-Gel effectively reverses the TGF-β1-induced fibrosis of NRK-52E cells via CS-mediated KLO secretion. Effect of free KLO on the mRNA expression of (A) TGF-β1, (B) α-SMA, and (C) fibronectin in NRK-52E cells. (D) Schematic diagram showing the mechanism by which TGF-β1 induces the activation of the TGF-β1/Smad2/3 signaling pathway, which is inhibited by KLO-Gel. (E) Representative Western blots showing the expression of RF-related proteins in NRK-52E cells exposed to the indicated treatment conditions. (F) CS restored endogenous KLO secretion in NRK-52E cells following TGF-β1 treatment. Data are expressed as the mean ± SD (*n* = 4). *P < 0.05, **P < 0.01, ***P < 0.001, ****P < 0.0001 versus the model group.

TGF-β1 is an RF-inducing cytokine, which promotes the differentiation of renal tubular epithelial cells into fibroblasts [32]. Therefore, the therapeutic effects of KLO-Gel were evaluated in TGF-β1-treated NRK-52E cells. Prior to these in vitro experiments, the expression of RF-related factors was determined in NRK-52E cells following TGF-β1 treatment. The fibrosis-related mRNA levels of TGF-β1 (Fig. 4A), α-smooth muscle actin (α-SMA) (Fig. 4B), and fibronectin (Fig. 4C) were significantly increased following a 48-h incubation with TGF-β1 (10 ng/ml). Conversely, the expression of these factors was down-regulated in the NRK-52E cells after exposure to an extremely low concentration (0.25 ng/ml) of free KLO. These results confirmed the potent anti-fibrotic properties of KLO as reported previously [7].

Likewise, the diluted KLO-Gel was incubated with the TGF-β1-treated NRK-52E cells. The TGF-β1/Smad2/3 signaling pathway plays a key role in RF, whereby Smad2/3 proteins are phosphorylated in response to TGF-β1 stimulation (Fig. 4D). As expected, TGF-β1 treatment indeed induced the phosphorylation of Smad2/3, which in turn induced myofibroblast activation and promoted extracellular matrix (ECM) accumulation in the NRK-52E cells (Fig. 4E). Interestingly, both KLO-Gel (containing 150 μg/ml CS and 0.25 ng/ml KLO) and BLK-Gel (containing 150 μg/ml CS) exerted stronger anti-fibrotic effects than free KLO. This was evidenced by KLO-Gel inhibited TGF-β1 and pSmad2/3 expression, improved fibroblast activation proteins E-cadherin and α-SMA, as well as decreased ECM protein fibronectin (Fig. S2).

To determine the mechanism by which KLO-Gel induced stronger anti-fibrotic effects than free KLO, the effects of CS on KLO production were determined in NRK-52E cells. After cotreated with TGF-β1 and CS (75, 150, and 300 μg/ml) in NRK-52E cells, the concentration of KLO in the cell supernatant was determined by ELISA. The KLO levels decreased after TGF-β1 treatment but increased following exposure to CS in a dose-dependent manner (Fig. 4F). These finding demonstrated that CS induced KLO secretion, explaining the significantly higher anti-fibrotic efficiency of KLO-Gel versus free KLO.

### In vivo thermosensitive gelation

To explore the injectability and in vivo thermosensitive gelation capacities of KLO-Gel, ink-loaded KLO-Gel was injected subcutaneously into the backs of normal mice. In vivo thermosensitive gelation was observed at 3 min after injection (Fig. S3A and B), indicating that KLO-Gel can form rapidly following local injection. In addition, the injectability and thermosensitive gelation of KLO-Gel in the kidney were evaluated by injecting 50 μl of ink-loaded KLO-Gel into the renal capsule of mice using a 30-gauge needle (Fig. S3C). Consistent with the subcutaneous injection experiment, the ink-loaded KLO-Gel achieved thermosensitive gelation in the kidney within 3 min. These results indicate that KLO-Gel can be effectively administered by local injection and undergoes thermosensitive gelation at the injection site in vivo.

### Safety and biocompatibility evaluation

**Fig. 5.**
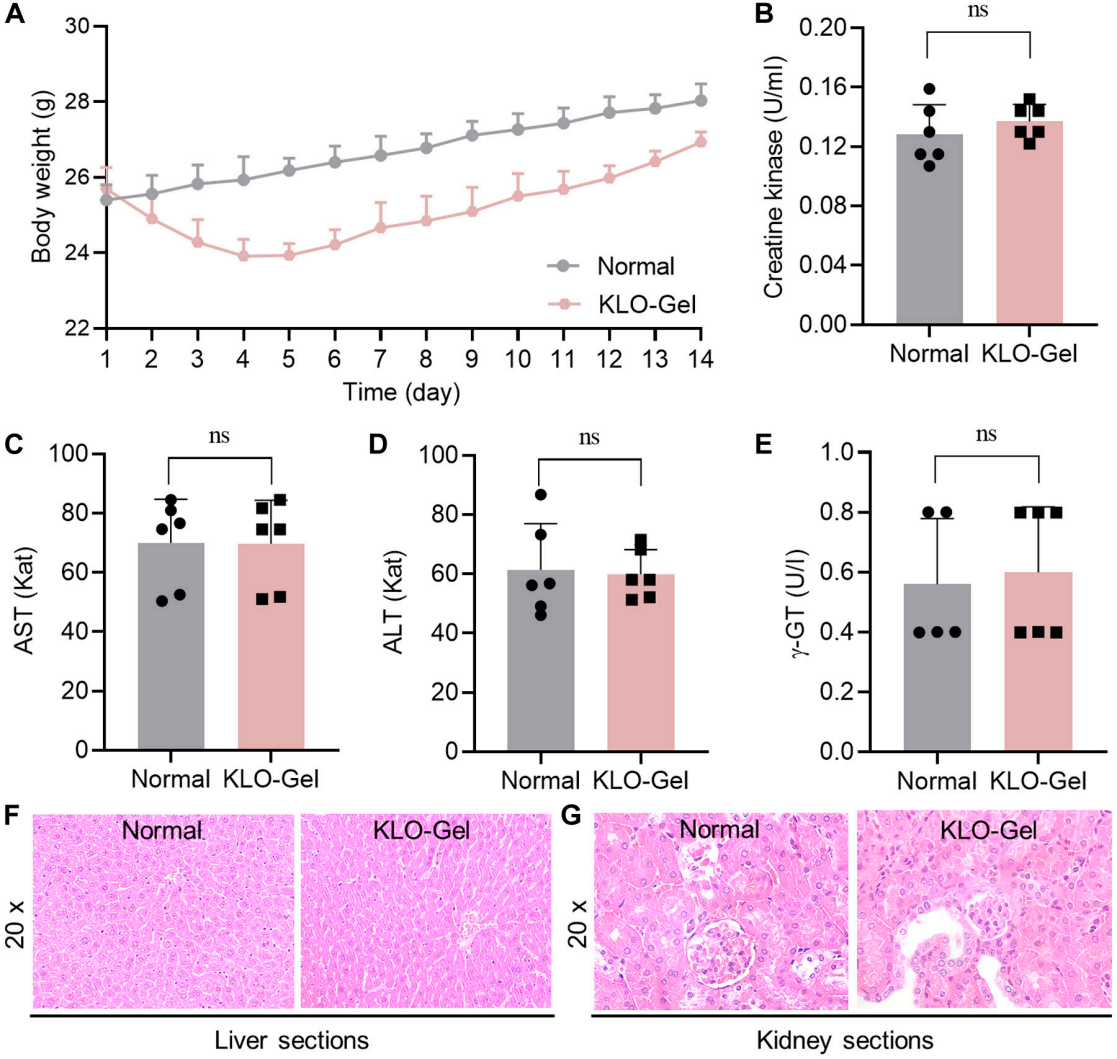
Safety and biocompatibility evaluation of KLO-Gel after localized renal capsule injection of mice for 14 d. (A) Body weight. (B) Serum creatine kinase levels and (C to E) liver enzyme activity parameters measured using commercial kits. Data are expressed as the mean ± SD (*n* = 6). There were no significant difference (ns) between the normal and KLO-Gel groups. H&E-stained (F) liver and (G) kidney sections at 20× magnification.

Before conducting the therapeutic efficacy studies, the safety and biocompatibility valuation of KLO-Gel were evaluated. The normal mice were locally injected with KLO-Gel in the kidney; age-matched mice that underwent a similar operation to expose their kidneys for injection but were not injected with KLO-Gel were used as normal control group. The body weights of KLO-Gel-treated mice increased steadily during the 14-d observation period (Fig. 5A). Compared to normal mice, no statistical change was detected in the levels of creatine kinase or liver enzyme activity parameters [aspartate transaminase (AST), alanine aminotransferase (ALT), and γ-glutamyl transferase (γ-GT)] of KLO-Gel-treated mice (Fig. 5B to E). Additionally, no histological changes were observed in the H&E-stained liver and kidney sections of KLO-Gel-treated mice versus in those of normal control mice (Fig. 5F and G). Collectively, these results demonstrate that KLO-Gel exhibited favorable safety and biocompatibility.

### In vivo anti-fibrotic efficacy

**Fig. 6.**
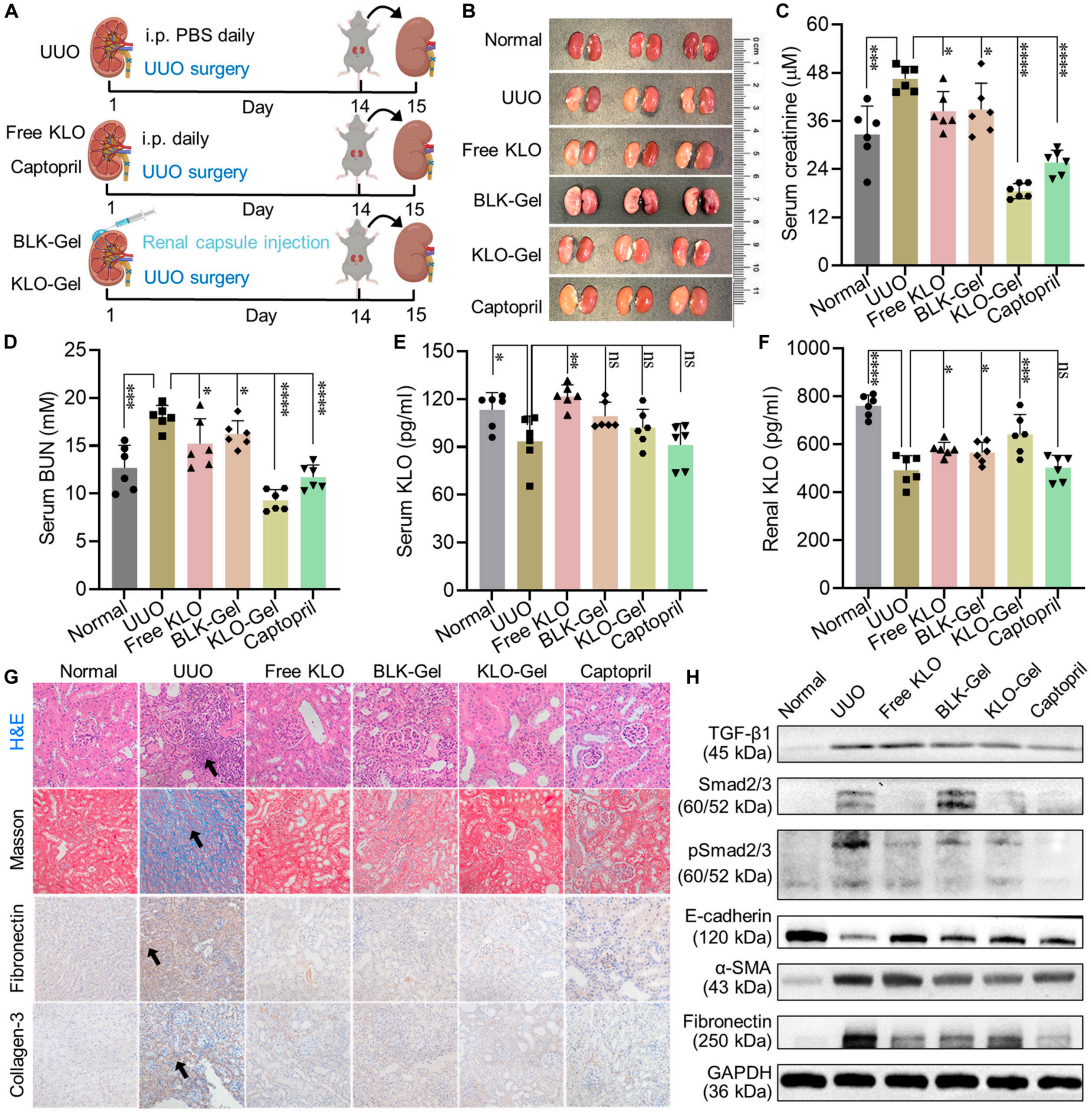
KLO-Gel alleviates RF in UUO mice by maintaining renal KLO levels. (A) Schematic illustration of treatment regimens. i.p., intraperitoneal. (B) Representative photographs showing the appearance of obstructed (left) and normal (right) kidney of UUO mice. Serum levels of (C) creatinine, (D) BUN, and (E) KLO after the administration of the indicated treatments. (F) Renal levels of KLO in the various treatment groups. (G) Histological sections (observed under 40× magnification) of the obstructed kidneys of UUO mice stained with H&E, Masson’s trichrome stain, and anti-fibronectin and anti-collagen-3 antibodies. (H) Representative Western blots showing the expression levels of the indicated fibrosis-associated proteins in the obstructed kidneys of UUO mice. Data are expressed as the mean ± SD (*n* = 6). *P < 0.05, **P < 0.01, ***P < 0.001, ****P < 0.0001 versus the UUO group.

The anti-fibrotic efficacy of KLO-Gel was evaluated in UUO mice. After UUO surgery, BLK-Gel (50 μl) and KLO-Gel (50 μl, containing 3.75 μg of KLO) were locally injected into the renal capsule of the mouse left kidney where they slowly released KLO over 14 d; meanwhile, free KLO (10 μg/kg/d) and captopril (10 mg/kg/d) were intraperitoneally injected to the mice once a day (Fig. 6A). On day 15, the kidneys of mice in all groups were collected and observed. Compared to the healthy (right) kidneys, the obstructed (left) kidneys in the UUO group showed typical pathological signs of RF, including atrophy, paleness, and poor elasticity (Fig. 6B). However, the obstructed kidneys in the KLO-Gel-treated group looked almost the same as the healthy kidneys, suggesting the robust anti-fibrotic efficacy. The KLO-Gel treatment also significantly reduced the serum creatinine and BUN levels (Fig. 6C and D), demonstrating that it effectively restored renal function.

In line with the kidney function results, the histopathology results confirmed that KLO-Gel significantly inhibited RF (Fig. 6G). KLO-Gel-treated mice exhibited less tubulointerstitial injury in H&E-stained sections (e.g., tubular epithelial cell apoptosis and inflammatory cell infiltration) than the mice in the free KLO and captopril groups. Furthermore, the KLO-Gel-treated mice had much less fibrous tissue (determined by Masson’s trichrome staining of kidney tissue sections) than the UUO mice, which confirmed that KLO-Gel significantly inhibited collagen accumulation and deposition. Immunohistochemical staining of kidney sections revealed that the KLO-Gel-treated mice had significantly fewer brown granules (fibronectin and collagen-3) than the UUO mice, implying that KLO-Gel alleviated ECM accumulation (Fig. S4).

To better understand the mechanism underlying the anti-fibrotic effects of KLO-Gel, we next determined the expression of proteins involved in the TGF-β1/Smad2/3 signaling pathway in the obstructed kidneys of UUO mice by Western blotting. As shown in Fig. 6H and Fig. S5, the elevated TGF-β1 levels in the UUO mice stimulated the phosphorylation of Smad2/3 and then induced myofibroblast activation, which were characterized by down-regulation of E-cadherin and up-regulation of α-SMA, thereafter resulting in high levels of fibronectin. However, these changes were effectively suppressed in all the treatment groups, especially for the free KLO-, KLO-Gel-, and captopril-treated groups. These results validated the potential of KLO-Gel as a promising treatment for RF.

To measure KLO concentrations in treated and untreated UUO mice, the serum and renal KLO levels were next determined by ELISA. Although the serum KLO levels were severely depleted in the untreated UUO group, they were significantly replenished following the daily injection of free KLO (Fig. 6E). Moreover, the serum KLO content of the free KLO group was significantly higher than that of all the other groups. Meanwhile, the renal KLO content of the KLO-Gel group was significantly higher than that of the free KLO and other groups, suggesting that KLO-Gel administration preferentially increased KLO levels in the kidneys.

## Discussion

KLO is an anti-fibrotic protein expressed in the kidneys. Despite showing promise as an anti-fibrotic agent in many preclinical studies [7], the systemic administration of recombinant KLO is often characterized by low kidney distribution and off-target toxicity, resulting in suboptimal therapeutic efficacy. Recombinant KLO has been predominantly administered via intraperitoneal injection, and few delivery systems have been developed to date for its targeted release in vivo. In one such study, an osmotic mini-pump was developed and implanted intraperitoneally to deliver KLO to CKD mice [33]. This osmotic mini-pump achieved a slow KLO release over 1 month, which markedly alleviated CKD and related pathologies in the animals. Alternatively, the localized delivery of therapeutic agents such as KLO could be used to achieve the desired therapeutic concentrations at the target site while minimizing systemic tissue toxicity. Thus, in the current study, we developed an injectable KLO-Gel and tested its ability to achieve the sustained release of KLO as a form of RF therapy.

In the formulation optimization studies, KLO-Gel prepared with CS:β-GP = 2:1 (v/v) achieved thermosensitive gelation within 3 min both in vitro and in vivo. In addition, the KLO-Gel formulation prepared using this CS to β-GP ratio exhibited a slow and sustained release in UUO mice over 14 d, tolerable levels of swelling, and degradation. Notably, in the in vitro anti-fibrosis efficacy studies using TGF-β1-treated NRK-52E cells, we showed that CS induced the secretion of KLO and enhanced the anti-fibrotic effects of KLO-Gel. These novel findings revealed the promising potential of CS in RF treatment. Thereafter, we demonstrated that KLO-Gel maintained the function and tissue structure of the kidneys, and mediated its anti-fibrotic effects by simultaneously increasing serum and renal KLO levels.

We acknowledge that our study had some limitations. First, although KLO-Gel demonstrated favorable safety during the 14-d observation period, long-term biosafety evaluation should be performed before it is considered in clinical applications. Second, the use of 14-d UUO model restricts our understanding of the therapeutic efficacy of KLO-Gel over a longer treatment period; thus, the anti-fibrotic efficacy of this reagents remains to be verified in a CKD mouse model. Third, despite that local injection of therapeutic agents into the renal capsule can be performed in the clinic [34,35], a less invasive route of KLO-Gel delivery should be investigated for use in patients.

In summary, an injectable KLO-Gel was developed for the localized and prolonged release of KLO in RF treatment. KLO-Gel achieved fast thermosensitive gelation and sustained release over 14 d in vitro and in vivo. KLO-Gel demonstrated a great potential in the management of RF by elevating renal rather than solely serum KLO concentrations in UUO mice. Overall, this locally injectable KLO-Gel represents a viable RF treatment strategy, which may be further explored for the management of CKD.

## Ethical Approval

Animal studies were performed in accordance with the National Research Council’s Guide for the Care and Use of Laboratory Animals and approved by the Animal Research Ethics Committee, Shenzhen University, Shenzhen, China (approval no. IACUC-202300106).

## Data Availability

The datasets used and analyzed during the current study are available from the corresponding author on reasonable request.
