# Supplementary material for: An In Situ Sustained-Release Chitosan Hydrogel to Attenuate Renal Fibrosis by Retaining Klotho Expression
Source: Biomater Res. 2024 Oct 24;28:0099. doi: 10.34133/bmr.0099 (PMC11499586; doi:10.34133/bmr.0099)
Supplement: Supplementary 1 — Figs. S1 to S5 Table S1 [file bmr.0099.f1.docx]

Supplementary Material

**An *in situ* sustained-release chitosan hydrogel to attenuate renal fibrosis by retaining klotho expression**

Chenyang Li ^1†^, Shuai Wang ^1†^, Chenghui Liao ^1^, Ying Li ^1^, Yunfeng Zhou ^2^, Haiqiang Wu ^1^, Wei Xiong ^1*^

^1^ School of Pharmacy, Shenzhen University Medical School, Shenzhen University, Shenzhen, 518055, China.

^2^ School of Basic Medical Sciences, Shenzhen University Medical School, Shenzhen University, Shenzhen, 518055, China.

^†^ These authors contributed equally to this work.

**Correspondence to:**

^*^ **Wei Xiong**

School of Pharmacy, Shenzhen University Medical School, Shenzhen University, 1066 Xueyuan Avenue, Nanshan District, Shenzhen, 518055, China

Tel: +86 0755 2691 3026

E-mail: [weixiong@szu.edu.cn](mailto:weixiong@szu.edu.cn)


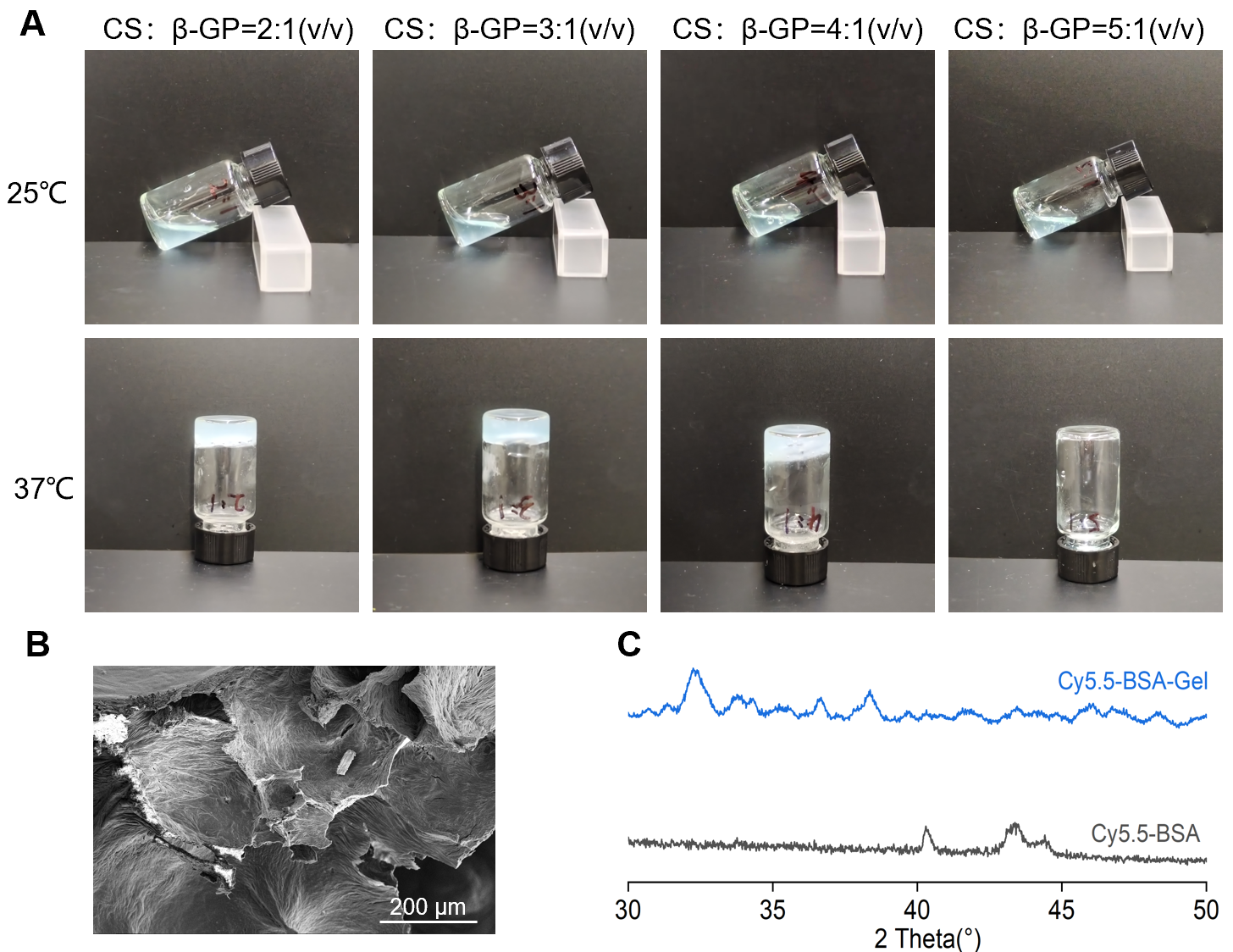


**Fig. S1.** *In vitro* characterization of Cy5.5-BSA-Gel. (A) Cy5.5-BSA-Gel was prepared using different chitosan (CS) to β-glycerophosphate (β-GP) ratios at 25°C, before undergoing thermosensitive gelation at 37°C. (B) Representative SEM image of lyophilized Cy5.5-BSA-Gel. (C) X-ray diffraction (XRD) patterns of Cy5.5-BSA-Gel.


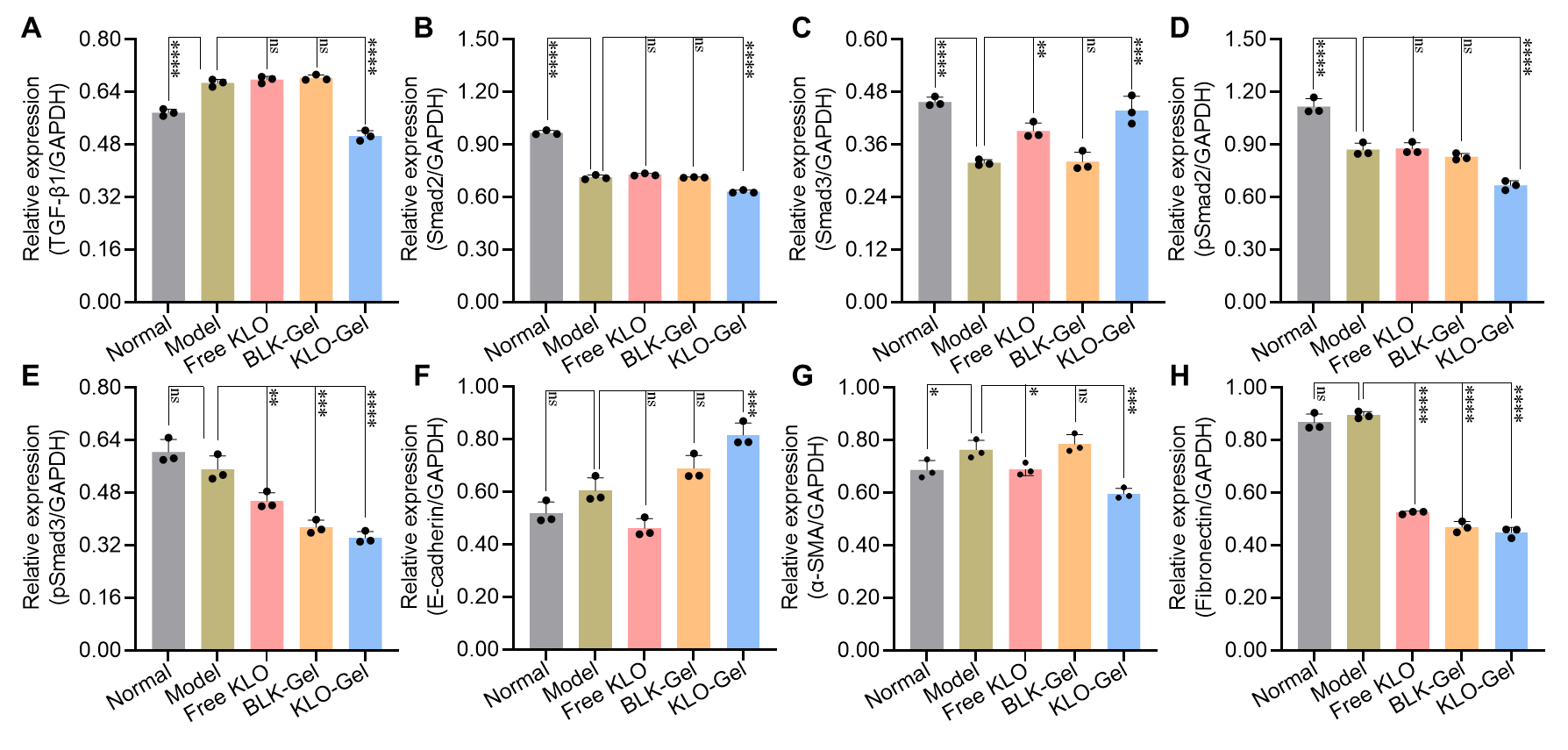


**Fig. S2.** The semi-quantitative analysis of protein expression in TGF-β1-treated NRK-52E cells. Data are expressed as the mean ± SD (n = 3), * *p<*0.05, ** *p<*0.01, *** *p<*0.001 and **** *p<*0.0001 *versus* the Model group.


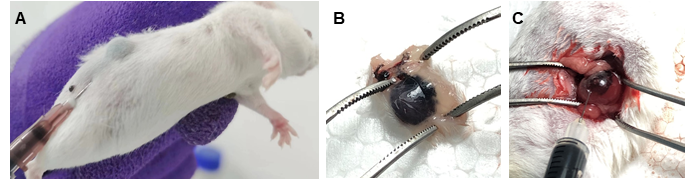


**Fig. S3.** *In vivo* thermosensitive gelation of ink-loaded KLO-Gel. (A) Ink-loaded KLO-Gel was injected subcutaneously into the back of normal mice. (B) Representative images of skin with ink-loaded KLO-Gel samples collected at 3 min post subcutaenous injection in mice. (C) Ink-loaded KLO-Gel was injected into the renal capsule of mice using a 30G needle.


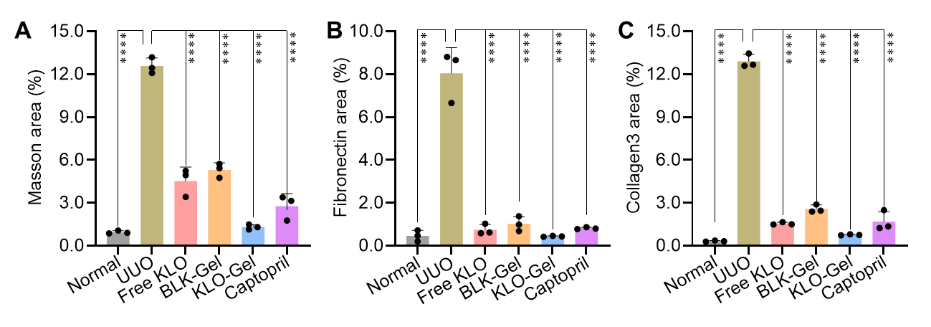


**Fig. S4.** Quantification of the percentage Masson, collagen-I and fibronectin accumulation. Data are expressed as the mean ± SD (n = 3), **p<*0.05, ***p<*0.01, ****p<*0.001 and *****p<*0.0001 *versus* the UUO group.


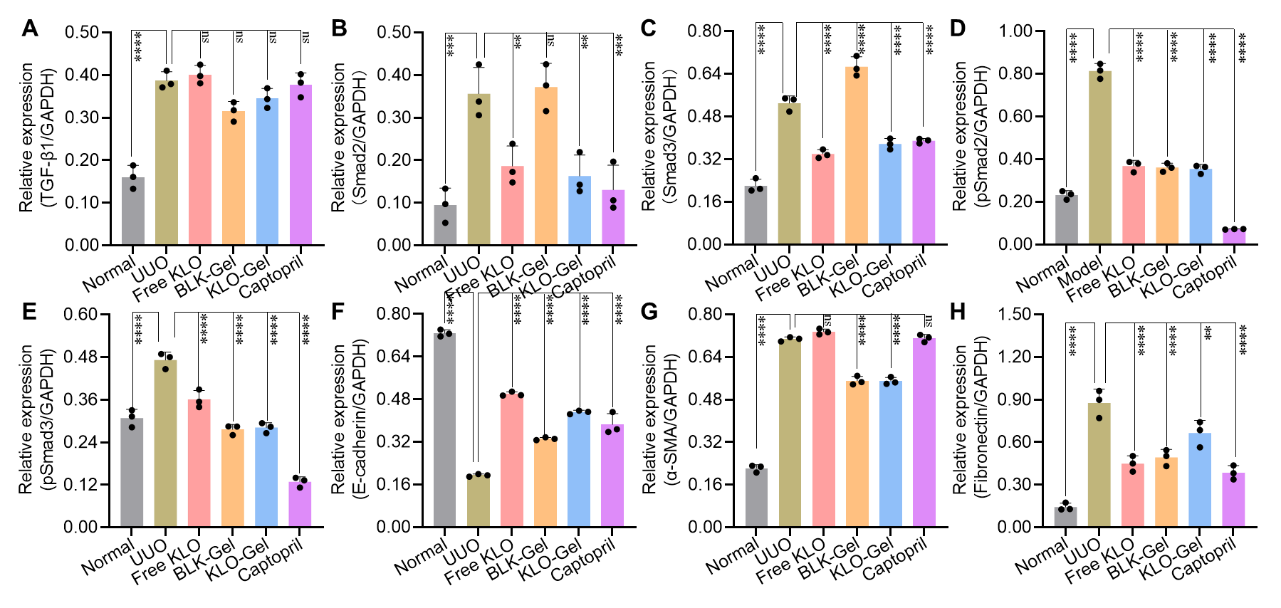


**Fig. S5.** The semi-quantitative analysis of protein expression in the kidneys of UUO mice after treatment. Data are expressed as the mean ± SD (n = 3), * *p<*0.05, ** *p<*0.01, *** *p<*0.001 and **** *p<*0.0001 versus the UUO group.

**Table S1.** The thermosensitive gelation of KLO-Gel at different temperature.

| Temperature | CS: β-GP=2:1 (v/v) | | CS: β-GP=3:1 (v/v) | | CS: β-GP=4:1 (v/v) | | CS: β-GP=5:1 (v/v) | |
| --- | --- | --- | --- | --- | --- | --- | --- | --- |
|  | 3 min | 5 min | 3 min | 5 min | 3 min | 5 min | 3 min | 5 min |
| 25℃ | × | × | × | × | × | × | × | × |
| 27℃ | × | × | × | × | × | × | × | × |
| 29℃ | × | × | × | × | × | × | × | × |
| 31℃ | ○ | ○ | × | × | × | × | × | × |
| 33℃ | ○ | √ | × | × | × | × | × | × |
| 35℃ | √ | √ | × | × | × | × | × | × |
| 37℃ | √ | √ | ○ | √ | × | × | × | × |
| Notes：√ means gelation, ○ means partial gelation, × means solution. | | | | | | | | |
